# Alkaline Reaction Pathways of Phenolic Compounds with β-Lactoglobulin Peptides: Polymerization and Covalent Adduct Formation

**DOI:** 10.3390/molecules30234584

**Published:** 2025-11-28

**Authors:** Alina Bock, Sarah Rottner, Daniel Güterbock, Ulrike Steinhäuser, Sascha Rohn, Helena Kieserling

**Affiliations:** 1 Department of Food Chemistry and Analysis, Institute of Food Technology and Food Chemistry, Technische Universität Berlin, Gustav-Meyer-Allee 25, 13355 Berlin, Germany; alina.bock@bht-berlin.de (A.B.); gueterbock@campus.tu-berlin.de (D.G.); helena.schestkowa@tu-berlin.de (H.K.); 2 Department of Food Technology and Food Analysis, Berliner Hochschule für Technik, Luxemburger Straße 10, 13353 Berlin, Germany; steinhaeuser@bht-berlin.de

**Keywords:** phenolic compounds, peptide, alkaline conditions, mass spectrometry, covalent adducts, oligomerization

## Abstract

A common strategy for a protein’s functionality modification is the covalent binding of phenolic compounds (PCs) under alkaline conditions. Whether intentionally applied or arising during food processing and storage, such reactions are highly relevant, as alkaline pH promotes oxidation, covalent adduct formation, and polymerization, thereby altering both PC and protein properties. However, the interplay of these reactions and the impact of PC structure remain insufficiently understood. This study aimed at characterizing covalent binding products of structurally related PCs with tryptic peptides of the model protein β-lactoglobulin (β-Lg) at pH 9. Emphasis was given on substitution patterns and steric effects influencing polymerization and peptide adduct building. Hydroxycinnamic acid and flavonoid derivatives differing in hydroxyl substitution and carrying polar (glycosidic) groups were selected. Incubation products were characterized by HPLC–DAD and high-resolution mass spectrometry. Results showed that both mono- and dihydroxy PC undergo oxidation under alkaline conditions, but with distinct reactivity. Monohydroxy PCs form only limited peptide adducts due to resonance stabilization and steric hindrance. In contrast, dihydroxy PCs displayed a higher reactivity, producing more polymerization products and covalent adducts. Their enhanced reactivity is linked to the ability of quinone formation with reduced electrostatic repulsion, while additional polar substituents promote interactions with polar amino acids. At the same time, these substituents impose steric constraints on PC polymerization, modulating oligomer size and thereby influencing peptide binding. Overall, the findings highlight structural determinants of PC reactivity and provide mechanistic insight into the balance between polymerization and covalent peptide modification under alkaline conditions.

## 1. Introduction

Proteins commonly interact with various food constituents, including phenolic compounds (PCs), through non-covalent and covalent interactions [1,2,3]. These interactions can alter both protein structure and functionality in food systems, thereby increasing the overall complexity of such systems. For instance, several studies showed that PCs modify protein secondary structures like β-sheets, α-helices, and unordered structures [4]. Such structural rearrangements, including partial unfolding, may alter surface hydrophobicity [5] and, consequently, functional properties such as interfacial adsorption, ultimately affecting the stabilization of food systems like emulsions and foams [6].

While non-covalent interactions generally induce minor or reversible structural changes, covalent bonds often result in more pronounced and irreversible structural rearrangements [7,8] that remain stable within a food matrix. The nature and extent of these covalent interactions depend on several factors like the chemical structures of the PC and the protein, as well as on reaction conditions such as the pH value. Especially under alkaline conditions and in the presence of oxygen, PCs can be oxidized to quinones via semiquinone intermediates, a pathway that promotes covalent adduct formation with nucleophilic protein side chains such as amino or thiol groups [1,9]. However, a competing pathway involves autoxidation, where reactive PC intermediates undergo radical–radical coupling, forming cross-linked products and initiating the formation of polymeric PC structures [1,10,11]. The latter include small oligomers, which can further react into higher-molecular-weight polymers. Notably, small PC oligomers, like dimers and trimers, can still react covalently with proteins [12,13], whereas larger PC polymers such as in tannins have been described to predominantly engage in non-covalent interactions with proteins [14].

As recently highlighted by Kieserling et al. (2024), the reaction dynamics of the formation of protein–PC interactions are still not comprehensively understood, as so many competing influences need to be considered [3]. Oxidation, PC oligomerization and polymerization, as well as protein binding obviously occur simultaneously and influence each other. Hence, understanding these reaction pathways is crucial, as the predominance of PC oligomerization or even polymerization, covalent protein binding, incorporation of oligomers and polymerization products, or non-covalent interactions ultimately determines functional outcomes in (protein-rich) food systems.

The various structural characteristics of PCs and proteins further complicate the elucidation of their reaction behavior. For PCs, the presence of dihydroxy structures (i.e., 1,2- or 1,4-dihydroxybenzene) facilitates quinone formation [15,16], while molecular size, flexibility, polarity and charge also influence their reactivity [17]. For proteins, the number and accessibility of nucleophilic side chains (e.g., lysine, cysteine and proline) are crucial. The interplay of these factors leads to highly dynamic and heterogenic mixtures, making it challenging to predict specific polymerization and protein–PC reaction products [3].

To further understand the course of these reactions and their interplay, it is therefore necessary to gain deeper insight into the individual influencing factors. Previous studies primarily analyzed protein–PC adducts using a wide range of structurally diverse animal and plant-based proteins, those pronounced differences in three-dimensional structures resulted in distinct steric limitations [2,17]. A systematic investigation of the influencing factors requires a reduced structural complexity to minimize interfering interactions. One effective approach is to reduce steric hindrance from protein secondary structures, thereby increasing the accessibility of potential PC binding sites. This can be achieved through enzymatic cleavage of proteins into peptides, which cannot maintain stable secondary structures, due to their shorter chain length. This strategy allows for a clear assessment of how the chemical structure of PCs governs covalent binding and oligomerization/polymerization, independent of steric constraints imposed by intact proteins.

Consequently, the aim of the present study was to investigate the interplay of the polymerization behavior and covalent adduct formation of structurally diverse PCs with peptides under alkaline conditions (pH 9). Peptides derived from the tryptic digestion of whey protein β-lactoglobulin were used as model systems. Specifically, this study focused on two interconnected aspects: (i) how different PC substitution patterns, including the number and the position of hydroxyl groups and the presence of polar moieties (e.g., glycosidic residues) influence the tendency of PCs to undergo oligomerization/polymerization, and (ii) how the chemical structure of PCs determines the extent and specificity of covalent bond formation with β-lactoglobulin peptides. Thereby, β-lactoglobulin is a quite prominent model protein, often used in model approaches, as it is fully structurally characterized. Its globular structure is helpful for studying conformation changes that might hint on interactions and reaction with other components [18,19].

Based on the chemical properties of the PC, it is hypothesized that monohydroxylated PCs generate only limited quinone intermediates under alkaline conditions due to insufficient resonance stabilization and electrostatic repulsion. Consequently, efficient free radical polymerization is hindered, and only minor amounts of oligomerization/polymerization products are formed. In contrast, dihydroxylated PCs are assumed to undergo polymerization more readily due to the absence of a permanent formal charge in the quinoid structure. PCs lacking polar substituents particularly favor radical–radical coupling reactions, as their binding sites are more sterically accessible. The resulting increased mesomeric stabilization lowers the redox potential, facilitating further polymerization. However, as polymer size increases, steric hindrance within the polymer structure limits subsequent interactions, ultimately suppressing further covalent binding. Covalent peptide binding is therefore expected to occur primarily in the early stages of oligomerization/polymerization, when reactive monomeric or low-molecular-weight oligomeric PCs are still available. Polar substituents can further promote reactivity by both sterically hindering polymer growth—preserving reactive species—and enhancing interactions with polar peptides via hydrogen bonding, thereby increasing the affinity for covalent bond formation. To test these hypotheses and characterize the various reaction pathways occurring under alkaline conditions, PCs and peptide–PC incubation products were separated chromatographically and analyzed using diode array detection (DAD) and high-resolution mass spectrometry (HRMS).

## 2. Results and Discussion

### 2.1. Characterization of β-Lg Peptides

**Figure 1:**
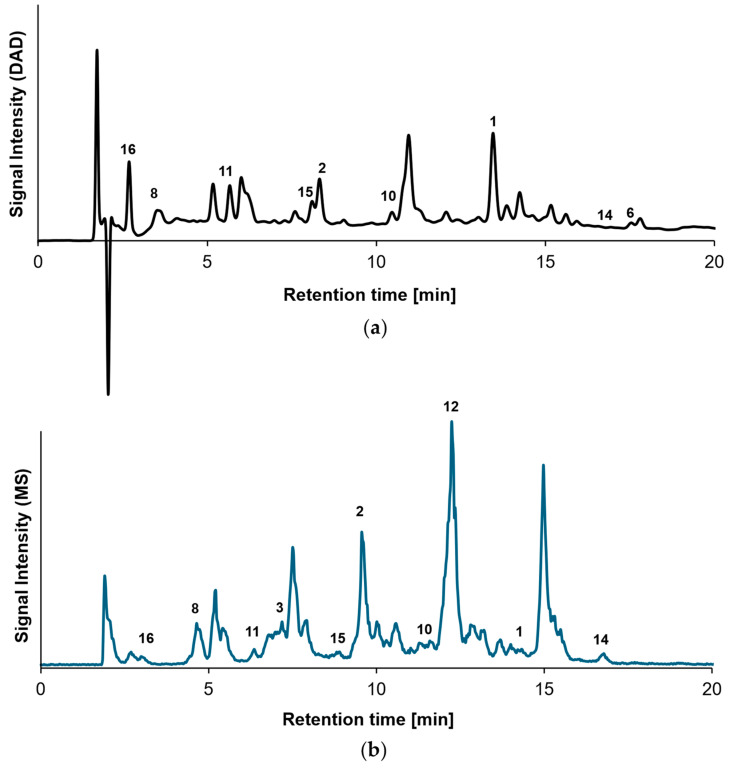
HPLC chromatograms of β-lactoglobulin peptides after tryptic digestion: (**a**) diode array detection (DAD) chromatogram (here at 205 nm), showing UV-absorbing peptides, and (**b**) total ion chromatogram (TIC) from mass spectrometry. The comparison illustrates the correlation between peptides detected by UV absorption and the overall ion signal. Peptide numbering corresponds to descriptions given in Table 1.

Following the tryptic digestion of β-Lg, the resulting peptides were separated chromatographically (Figure 1) and the mass spectrometric signals recorded at distinct retention times were assigned to their corresponding amino acid sequences (Table 1). Under the applied chromatographic conditions, a total of 19 peptides were effectively separated. Free lysine residues, however, due to their small size and high polarity, exhibited insufficient retention. All MS peaks corresponding to peptides with sufficient UV activity were successfully assigned to DAD peaks, except for peptides No. 3, 4, 5, 17, 18, and 19 (Table 1), whose low UV activity did not allow for comprehensive DAD signals. Systematically, retention times determined by MS and DAD differed by approximately 0.9 min.

The chemical properties of the peptides were characterized using the Grand Average of Hydropathicity (GRAVY) value, the theoretical isoelectric point (IEP), and the molecular weight (MW), as these parameters critically influence peptide interactions with PCs. GRAVY is a numerical measure of the overall hydropathic nature of a peptide, with negative values indicating hydrophilicity and positive values indicating hydrophobicity. Both properties determine the binding affinity to hydrophobic regions of PCs [20,21]. The IEP provides information on the net charge of a peptide as a function of pH, thereby allowing the evaluation of its electrostatic interactions with ionizable phenolic groups. Molecular weight relates to steric factors that can modulate the accessibility and stability of peptide–PC complexes. In fact, peptides No. 1, 2, 4, 8, 10, 12, 14, and 18 (Table 1) exhibited positive GRAVY values, reflecting their predominantly hydrophobic character and a high content of hydrophobic amino acids.

The IEP of a peptide can be used to estimate its net charge under the specific pH conditions. Most of the peptides incubated at pH 9 have usually an IEP < 9, which results in a negative net charge [22]. This can often be attributed to the presence of carboxyl groups in amino acid residues such as aspartic acid or glutamic acid, which are deprotonated—and thus, negatively charged—even at weak acidic pH conditions [23]. Peptides No. 2, 10, and 17 are nearly neutral at pH 9, despite containing lysine residues. At this pH value, the ε-amino groups of lysine remain protonated and hence, are positively charged, but their effect is balanced by negatively charged residues within the peptide sequences, resulting in an overall neutral net charge [24].

The MW of the peptides range from *m*/*z* 146.19 for the individual amino acid lysine up to *m*/*z* 2708.08 for peptide No. 4. Smaller peptides, such as peptides No. 2, 3, 6, 7, 8, 9, 10, 11, 12, 13, 15, 16, 17, and 18, with molecular weights up to approx. *m*/*z* 1250, often exhibit increased molecular flexibility and conformational dynamics compared to larger peptides. This is due to their limited possibility to form intramolecular hydrogen bonds and their general inability to form highly ordered secondary structure elements. According to Löwik & van Hest (2004), the formation of ordered β-sheet structures typically requires peptide lengths of at least 15 amino acids [23]. Nonetheless, in some cases, shorter sequences may adopt β-hairpin-like conformations, particularly, when alternating hydrophobic and hydrophilic residues are present [23]. This increased structural flexibility of peptides, resulting from a diminished secondary structure elements compared to high-molecular-weight proteins, can enhance the steric accessibility of binding sites for covalent interaction partners, thereby, potentially increasing the peptides’ reactivity towards PCs. At the same time, peptide size correlates quite well with solubility in water; larger peptides are more prone to aggregation and precipitation [25], which could reduce the availability of binding sites for an interaction with PCs. However, under the incubation conditions at pH 9, where many functional groups are deprotonated and negatively charged, all studied peptides remained in solution, supporting a potential reactivity towards PCs.

### 2.2. Polymerization Behavior of Monohydroxy PCs

**Figure 2:**
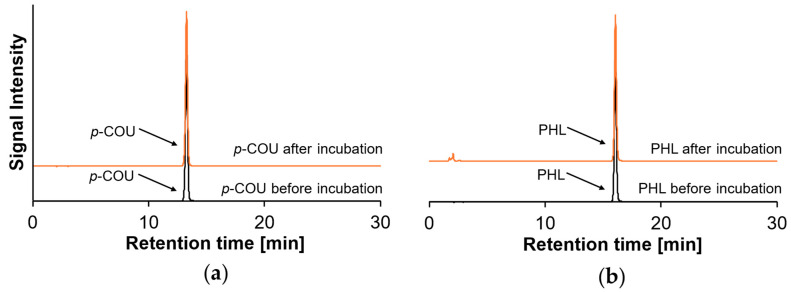
HPLC–DAD chromatograms (here at 315 nm) of (**a**) *p*-COU before (black line) and after (after line) incubation, and (**b**) PHL before (black line) and after (orange line) incubation.

The oligomerization/polymerization behavior of the monohydroxylated PC *p*-COU and PHL in the absence of peptides was characterized. Studying PC polymerization individually provides a baseline to understand how competition with nucleophilic peptide residues may shift the balance between polymerization and covalent adduct formation, a key aspect of the dynamic reaction pathways. As shown in Figure 2, both PCs exhibit a single peak with unchanged retention time before and after incubation, indicating that no detectable covalent reactions or polymerization products could be observed under the applied reaction conditions.

Although oxidative reactions of monohydroxy PCs involving phenoxyl radicals via single-electron transfer have been reported [26,27], the chromatographic results in Figure 2 indicate that no such reactivity occurs under the present conditions. While Thi et al. (2008) demonstrated that targeted polymerization of *p*-COU is possible, their experiments involved potentiodynamic electrolysis at high temperatures of 200 °C under strongly alkaline conditions [28]. These experimental conditions highlight the substantial energetic input needed to initiate oxidation of *p*-COU via phenolate electron abstraction. In contrast, the far milder conditions used in the present study (pH 9, 20 °C) likely do not provide a sufficiently oxidative environment to induce *p*-COU polymer formation. An alternative oxidation pathway of *p*-COU was demonstrated by Antolovich et al. (2004), who applied Fenton oxidation using Fe(II) and H_2_O_2_ [27]. Rather than promoting polymerization, this reaction yielded a cleavage product—4-hydroxybenzaldehyde. However, this degradation product was not detected under the (chromatographic) conditions used in the present study. Other, more recent studies confirm that oligomerization and polymerization of PCs is highly structure-dependent. Zhang et al. (2022) showed that controlled polymerization of diverse PCs can be achieved by modifying their chemical structures [29]. Eugenol, a monohydroxylated PC, is unable to undergo efficient radical-mediated polymerization due to the low reactivity of its allyl double bond and the radical-scavenging activity of its phenolic hydroxyl group. Various chemical modifications yielded in a higher reactivity of eugenol monomers, including methacrylation or allylation.

**Figure 3:**
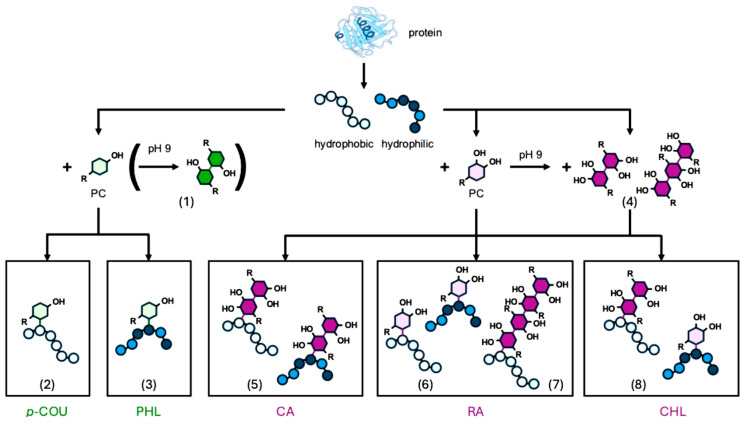
Illustrated overview of reaction patterns of PCs with and without β-Lg peptides at pH 9. (1) Dimerization of monohydoxy PCs, (2) covalent binding of *p*-coumaric acid (*p*-COU) with hydrophobic peptides, (3) covalent binding of phlorizin (PHL) with hydrophilic peptides, (4) oligomerization and polymerization of dihydroxy PCs, (5) covalent binding of caffeic acid (CA) and its oligomerization products with hydrophobic and hydrophilic peptides, (6) covalent binding of rosmarinic acid (RA) with hydrophobic and hydrophilic peptides, (7) covalent binding of RA oligomerization products with hydrophobic peptides, and (8) covalent binding of chlorogenic acid (CHL) with hydrophilic peptides and its oligomerization products with hydrophobic peptides.

Regarding PHL, Ikumi et al. (2008) demonstrated that its polymerization under alkaline conditions is indeed feasible; incubation at 90 °C resulted in the formation of PHL polymers with molecular weights up to 57,800 g/mol, although this required the presence of ω-amino-triethylene glycol as a linking agent [30]. These findings underscore that elevated temperatures enhance the reaction kinetics. Alternatively, oxidative polymerization of PHL can be promoted enzymatically, as shown by Zhou et al. (2021), who used laccase to generate phenoxyl radicals on the B-ring of PHL, thereby initiating polymer formation [31]. In contrast, the incubation conditions applied in the present study appear insufficient to induce oxidation or polymerization of PHL. The limited formation of semiquinones from PHL, owing to its monohydroxy structure, results in a restricted number of electrophilic intermediates during incubation, thereby hindering efficient radical polymerization (c.f. Figure 3, reaction product No. 1).

### 2.3. Reaction Behavior of Monohydroxy PCs with β-Lg Peptides

**Figure 4:**
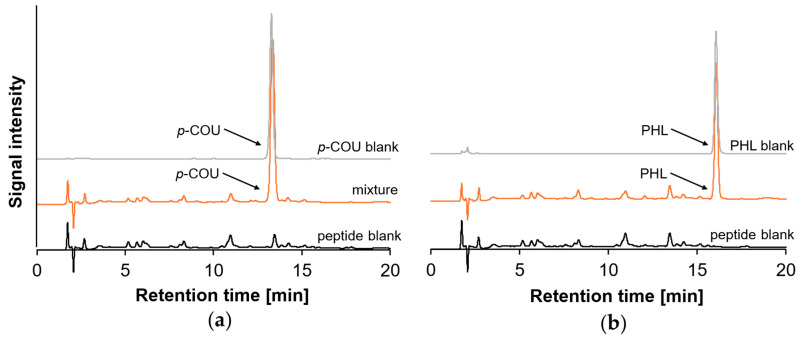
HPLC–DAD chromatograms (here at 315 nm) of pure PC (blank), β-Lg peptides before (black) at 205 nm and after incubation (orange) at 205 nm with the phenolic compounds *p*-COU (**a**) and PHL (**b**).

Following the observation that monohydroxy PCs do not typically form oligomerization/polymerization products upon incubation at pH 9, subsequent experiments were performed in the presence of peptides. The β-Lg peptides were likewise incubated with *p*-COU and PHL at pH 9. Analysis of the DAD chromatograms of the peptides before and after incubation with *p*-COU and PHL (Figure 4) revealed no differences in the peptide peaks with respect to retention time or peak area. Solely peaks with high signal intensity corresponding to unreacted *p*-COU and PHL were detectable, alongside a few additional peaks at lower retention times attributed to the peptides.

In contrast to the HPLC–DAD results (Figure 4), MS data (Table 2 and Table 3) for the incubated peptides revealed the presence of higher masses compared to the masses of the initial peptides. These shifts in molecular weights indicate the formation of distinct covalent peptide–PC adducts for both *p*-COU and PHL. The absence of noticeable changes in the DAD chromatograms suggest that this method may lack sensitivity to detect the relatively low concentrations of the formed adducts or an alteration in UV activity.

Covalent binding between proteins and monohydroxy PCs has been demonstrated in various model systems. One frequently referenced example is the reaction of ferulic acid with whey proteins, where lysine and tryptophan side chains have been identified as predominant sites of modification [32]. Under alkaline conditions and in the presence of atmospheric oxygen, monohydroxy PCs can undergo oxidation to from semiquinone radicals via electron transfer to oxygen [15]. However, this process is energetically less favorable compared to PCs with multiple hydroxyl groups, which enable resonance stabilization of the radical intermediate. As a result, semiquinone formation is less likely for monohydroxy PCs than for more resonance-stabilized systems such as *o*-dihydroxy compounds. Furthermore, semiquinones tend to form dimers rather than reacting with other nucleophiles. For example, ferulic acid can undergo oxidative coupling to yield dehydrodimers such as 8–5′ and 8–*O*–4′ linkages in both alkaline and enzymatic oxidation systems [33,34], thereby reducing its potential for covalent interaction with peptides. In the present study, however, no such putative dimeric species were detected based on exact mass (c.f. Section 3.2), indicating either their absence or formation below the detection threshold of the applied analytical methods. The limited extent of radical formation and subsequent covalent reactions with other nucleophiles may explain the absence of detectable peptide–PC adducts in the HPLC–DAD analysis (typical detection limit: 10^−6^–10^−9^ g), whereas the same adducts can be identified using the more sensitive HPLC–MS (typical detection limit: 10^−12^–10^−15^ g).

The detected adduct masses between the peptides and *p*-COU are summarized in Table 2.

Notably, the peptides forming adducts with *p*-COU—specifically peptides No. 2, 4, 8, 10, 12, and 14—are predominantly non-polar in character (Figure 3, reaction product No. 2), although each contains at least one positively charged lysine residue. The pKa value of the phenolic hydroxyl group in *p*-COU is approx. pKa2 = 9.0 [35], which closely matches the incubation conditions of pH 9.0. At this pH value, deprotonation of the phenolic hydroxyl groups of *p*-COU begins, resulting in a negative charge. This enables *p*-COU to engage in ionic interactions with positively charged lysine residues, as well [36]. Furthermore, the presence of an aromatic ring with an aliphatic substituent gives *p*-COU a conjugated π-electron system, which may favor hydrophobic interactions and π-stacking [15,37]. These non-covalent interactions could orient the molecules in a configuration conducive to covalent reactions; however, it should be emphasized that the structural implications remain tentative and have not been directly confirmed.

The adduct formation observed during the incubation of peptides with PHL follows a similar pattern to that of the adducts formed with *p*-COU (Table 3).

Similar to *p*-COU, adducts with PHL were detected only in the MS chromatogram, indicated by shifts in retention times and increases in molecular masses (Table 3). Overall, fewer peptides covalently reacted with PHL compared to *p*-COU. These findings support the hypothesis that PHL forms covalent adducts with β-Lg peptides only to a very limited extent, consistent with its lower overall reactivity.

The low reactivity of PHL can be attributed to its chemical structure, which is less prone to reacting with nucleophilic amino acid than comparatively more reactive PC such as *p*-COU or others. Unlike *o*-dihydroxy PCs that can be oxidized to reactive quinones capable of forming covalent bonds to amino or sulfhydryl groups in peptides, PHL also lacks this structural feature and predominantly engages in non-covalent interactions, such as hydrogen bonds [34]. These non-covalent interactions are typically assumed to be the basis for a subsequent covalent reaction. However, PHL exhibits a relatively low binding affinity for various proteins through non-covalent interactions when compared to larger PCs such as the flavonols quercetin or kaempferol as shown by Nagy et al. (2012) [35]. This limited ability to form stable non-covalent interactions may further explain the observed low extent of covalent adduct formation.

Additionally, steric hindrance can impede the interaction between PHL and peptides. The relatively rigid structure of PHL, along with the sterically hindering glucoside group at the A-ring, as described by Wei et al. (2024), limits access to potential binding sites of peptides, thereby, further reducing the extent of covalent adduct formation [38]. Such reduced adduct formation has been previously demonstrated by Cao et al. (2019), who showed that glycosidic groups of PCs can block potential binding sites, e.g., in plasma proteins [39]. As a result, the restricted accessibility limits direct covalent binding and also, reduces the probability of polymerization, further decreasing the overall reactivity of PHL toward peptides.

This pattern may be attributed to the polar glycosidic moiety of PHL, which may promote hydrogen bonding with amino acid side chains bearing hydroxyl, amino, or carboxyl groups. A similar interaction pattern was recently observed for other glycosylated phenolic acid derivative, such as verbascoside [40]. Hydrogen bonds promote the association of PHL with highly polar peptides, which are rich in hydrophilic substituents capable of forming hydrogen bonds.

Furthermore, PHL exhibits only low hydrophobicity (LogP = 0.98, calculated using Cxcalc (ChemAxon Ltd., Budapest, Hungary), which may limit its tendency to form adducts with hydrophobic peptides such as peptides No. 1, 12, and 14. Nevertheless, adduct formation with these nonpolar peptides was still observed (Table 3), potentially facilitated by ionic interactions between the deprotonated hydroxyl group of PHL and positively charged, protonated lysine residues. These interactions may increase binding affinity and slightly enhance the likelihood of covalent bond formation as described by Shahidi & Dissanayaka (2023), even in the absence of strong hydrophilic or hydrogen bonding interactions [7].

### 2.4. Polymerization Behavior of o-Dihydroxy PCs

**Figure 5:**
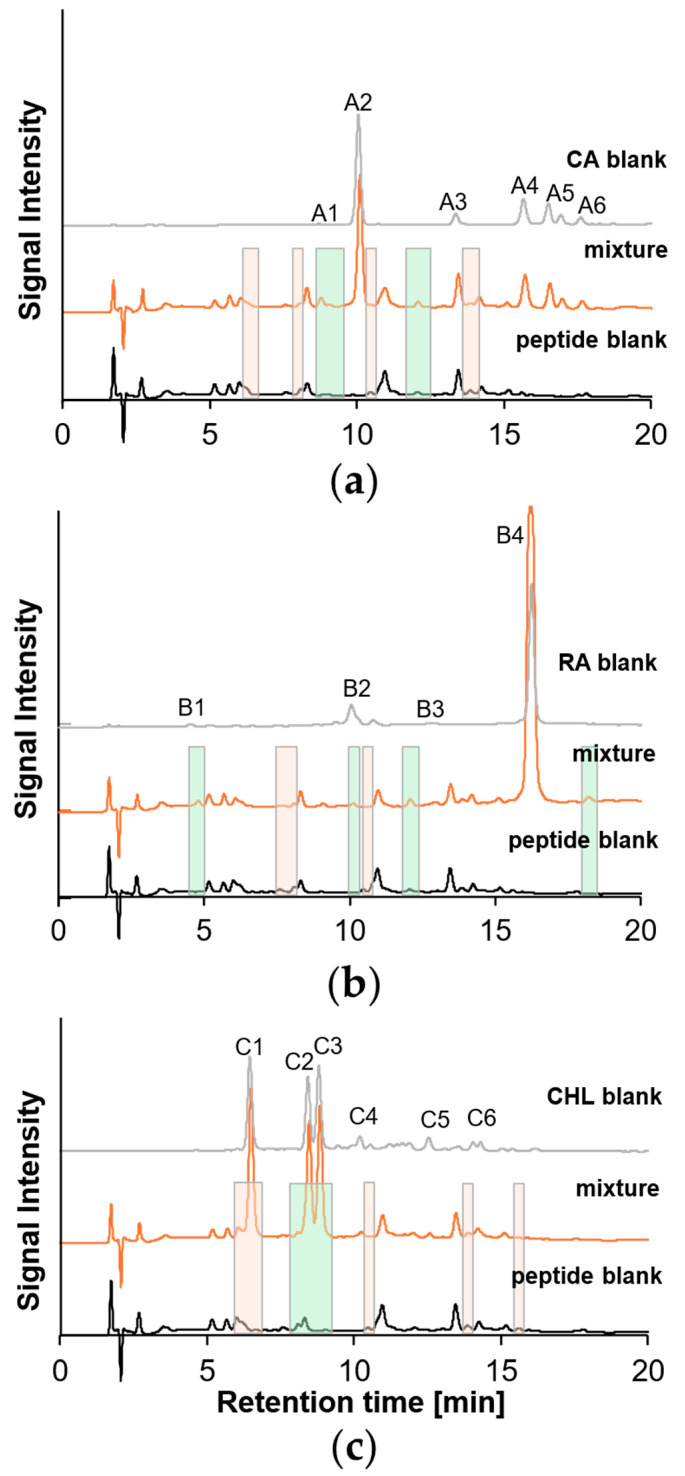
DAD chromatograms of β-Lg peptides before (peptide blank: black line) at 205 nm and after incubation with PC (mixture: orange line) at 205 nm and the incubated PC without peptides (“PC” blank: gray line) for the PC (**a**) CA, (**b**) RA, and (**c**) CHL at 315 nm. Orange boxes indicate alterations in the chromatogram characterized by a decrease in the signal intensity of the pure peptide peaks. Green boxes indicate changes in the chromatogram manifested as the formation of new peaks upon incubation. Explanations of peaks A1–A6 can be found in Table 4, peaks B1–B4 in Table 5, and peaks C1–C6 in Table 6.

Initially and to act as a kind of control, the reactivity of *o*-dihydroxy PCs was investigated to assess their potential oligomerization/polymerization behavior in the absence of peptides. As expected, the results showed that PCs with *o*-dihydroxy structures undergo autoxidation, reacting with themselves under oxidative conditions to form brown, high molecular weight polymerization products (Figure 3, reaction products No. 4). Table 4 presents the molecular masses of these oligomerization/polymerization products for CA, which exhibit high signal intensity in both DAD (Figure 5a, CA blank) and MS detection.

It is well-accepted that dihydroxy PCs can readily undergo oxidative transformations, resulting in the formation of quinones [3]. After the 16 h incubation, CA has not completely reacted, as its monomeric quinone form was still detectable (Table 4, peak A2). In addition, five new peaks appeared in the DAD and MS chromatograms, indicating the formation of putative dimeric CA species based on exact mass (Table 4, peaks A2, A3, A4, and A6). Previous studies suggest that the specific structure of such CA dimers dependent on reaction conditions such as pH, oxygen content, and the presence of catalysts.

**Figure 6:**
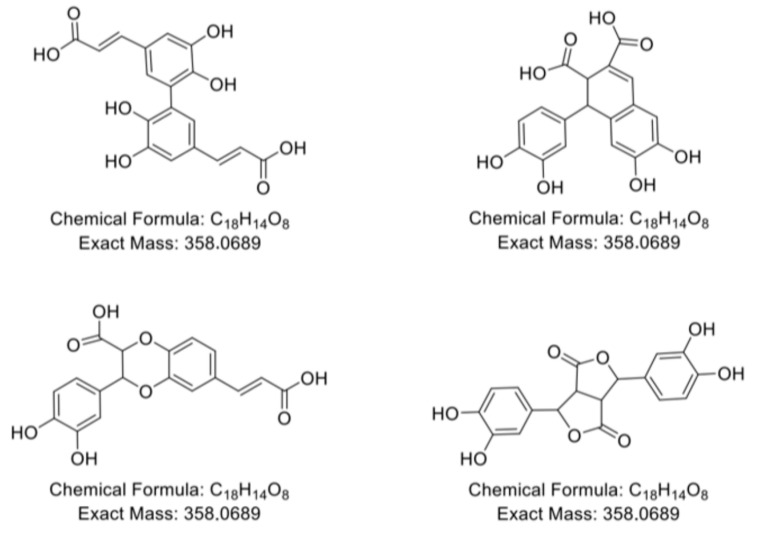
Chemical structure of possible CA dimers according to [13,41].

Likewise, in the present study, the variety of peaks corresponding to the molecular weight of dimeric CA reflects the formation of various isomeric adducts previously described in the literature [13,41]. Selected examples of these isomeric CA dimers formed under oxidative conditions are illustrated in Figure 6.

As emphasized by Kieserling et al. (2024), such putative dimers represent only a fraction of the structurally diverse oligomerization/polymerization products generated during oxidative PC coupling [3]. The coupling of CA radicals can proceed through multiple pathways, yielding positional isomers that differ in the linkage type and substitution pattern. These low-molecular-weight adducts can undergo further oxidative transformations, generating branched or cyclic oligomers that may serve as intermediates in higher-order network formation and potential crosslinking reactions. Such sequential coupling reactions can extend beyond the dimer stage, favoring the formation of higher oligomers under favorable conditions.

In addition to the observed CA dimers, CA trimers and hexamers were also detected, though with lower signal intensities (Figure 5a and Table 4). The formation of such higher molecular weight polymerization products of CA has previously been reported [16]. According to Hapiot et al. (1995), the reaction conditions influence whether predominantly dimers, oligomers, or even larger polymeric structures are formed [26]. Oligomerization is proposed to proceed via oxidation of the CA phenolate to a phenoxyl radical, which subsequently dimerizes through radical–radical coupling, a process that occur even under low-energy reaction conditions [26]. In contrast, CA trimer formation has been associated with high-energy conditions, such as the application of electrochemical potentials above 1.0 V in an alkaline environment [41]. These findings suggest that trimer formation of CA requires more specific or higher-energy conditions than dimerization.

The chromatograms of incubated RA (Figure 5b, RA blank) differ notably from those of the incubated CA. Only two peaks with high signal intensity were detected, one of which—at approx. 17 min—corresponds to the retention time of monomeric RA (Table 5, peak. B3). However, various polymerization and decarboxylation products of RA were also identified, many eluting at the same retention time as the monomer (Table 5, peak B3). This overlap indicates limited chromatographic separation between monomeric, oligomeric and polymeric RA.

**Figure 7:**
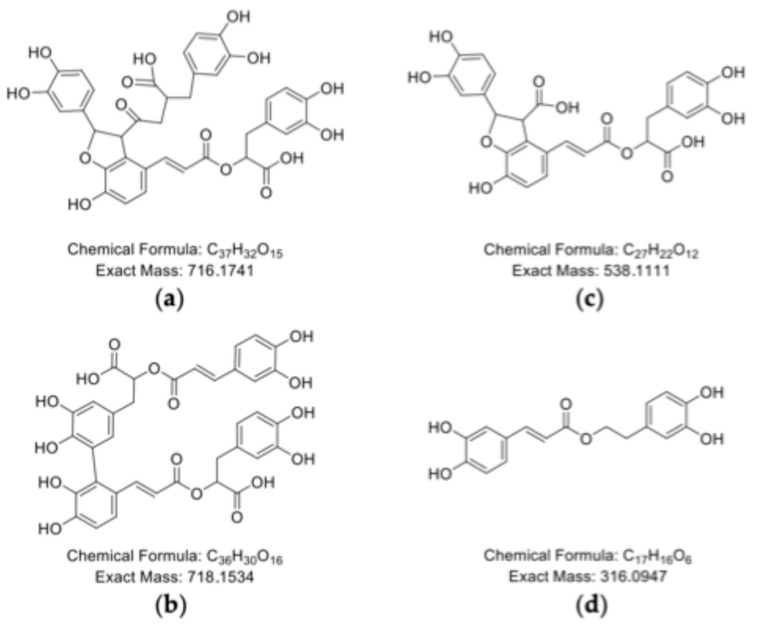
Chemical structures of possible RA oxidation products according to [42,43]. (**a**,**b**) represent possible dimeric structures of RA, (**c**) is a reported oxidation product derived from RA and CA, and (**d**) represents a potential decarboxylation product of RA named teucrol.

Among the identified oligomerization products of RA, putative dimers, trimers, tetramers, and pentamers were assigned based on exact mass, indicating a tendency of RA to form higher-molecular-weight species compared to CA. The formation of such RA dimers and trimers has previously been demonstrated by Ly et al. (2006) [42]. Those authors described the dimer as a potential condensation product of two RA molecules formed via oxidative cyclization, resulting in a 2,3-dihydrobenzofuran ring structure (Figure 7). Ly et al. (2006) [42] also elucidated two RA trimers. In one of these trimers, the conjugated olefinic bond of the CA moiety from the first RA molecule binds to the benzene ring of the 3,4-dihydroxyphenyl lactic acid unit of the second RA molecule, while the olefinic bond of the CA moiety of the third RA molecule is then attached to the benzene ring of the 3,4-dihydroxyphenyl lactic acid unit of the first RA molecule. In the other trimer, the olefinic bond of the first RA molecule connects to the benzene ring of the CA unit of the second RA molecule, while the third molecules’ olefinic bond links to the lactic acid unit of the second RA molecule [42].

The generally low intensities of the DAD peaks and the MS signals of RA polymers can be attributed to their decreasing solubility with increasing molecular weight. This solubility limitation could be further emphasized, as higher-molecular-weight RA oligomers are prone to aggregation and precipitation, reducing their detectability in solution-based analyses. Consequently, it is assumed that most RA polymers are removed during membrane filtration or are bound in the chromatographic pre-column. The precipitated solid could be visually observed in the incubation vessel, which was not the case for the other PC. These findings suggest that the additional 3,4-dihydroxyphenyl lactic acid unit in RA may enhance its tendency to form higher-molecular-weight species compared to CA at pH 9.

In addition to oligomerization/polymerization products, a decarboxylation product of RA—identified as teucrol [43,44]—was also detected. However, as decarboxylation reactions typically require elevated temperatures of approx. 100 and 200 °C [45], it is assumed that this reaction occurred only to a limited extent under the incubation conditions applied in the present study. Thus, polymerization of RA appears to have been the predominant reaction pathway.

**Figure 8:**
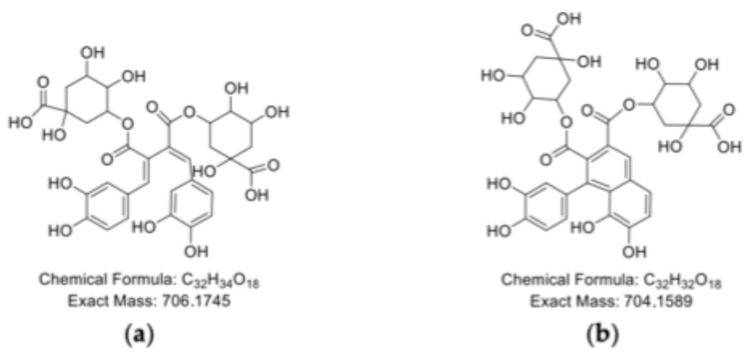
Chemical structure of potential CHL oxidation products according to [48], (**a**) non-cyclic dimeric structures of CHL, and (**b**) cyclic dimeric structures of CHL.

The chromatograms of incubated CHL (Figure 5c, CHL blank) differ markedly from those of the incubated CA and RA, showing a broad range of peaks between 7 and 17 min in the DAD chromatogram. Three of these peaks exhibited particularly high signal intensity (Table 6, peak C1 and C2) and were identified via mass analysis as monomeric CHL. The presence of multiple monomeric peaks may be attributed to isomerization processes, in which 5-CHL convert into its 3- and 4-isomers. These reactions are known to be initiated at pH 6 and intensify with increasing pH [46,47], suggesting a high isomerization rate under the applied alkaline conditions in this study. Peaks with lower signal intensity predominantly correspond to CHL dimers, which may also occur in cyclic forms (Figure 8), accordingly [47,48].

As no higher-molecular-weight CHL polymerization products were detected after the incubation, consistent with the findings of Prigent et al. (2008), it can be assumed that CHL dimers represent a thermodynamic state that does not favor further polymerization [48].

### 2.5. Reaction Behavior of o-Dihydroxy PCs with β-Lg Peptides

To investigate the reactivity of PCs containing *o*-dihydroxy structures in the presence of β-Lg peptides, incubations were carried out using CA, RA, and CHL. Figure 5a shows the DAD chromatogram from the CA–peptide incubation, alongside reference chromatograms of the peptides and individually. The peak at approx. 10 min corresponds to monomeric CA (Figure 5a, peak A2). Compared to the blanks, the chromatogram of the CA–peptide mixtures reveals distinct changes in peak patterns. Several signals around 6, 8, 10, and 14 min markedly decrease or disappear (Figure 5a, orange boxes), indicating chemical modification of the peptides. In addition, new peaks appear at retention times around 9 and 12 min (Figure 5a, green boxes), suggesting the formation of CA–peptide adducts. Additional peaks labeled A1 to A6, correspond to CA oligomerization/polymerization products (c.f. Section 3.4), confirmed by comparison with the CA blank (Figure 5a).

Additionally, HPLC–MS was performed with the CA-incubated peptide samples to identify specific adduct masses. Table 7 lists the detected CA–peptide adducts formed during incubation. As already observed in the DAD chromatogram (Figure 5a), adduct formation results in shifts in peptide retention times, which in some cases span relatively large windows (>5 min). While we interpret these shifts as indicative of covalent adduct formation, other explanations, such as co-elution or in-source clustering, cannot be completely excluded. Both the original retention time of the peptides and those of the corresponding adducts are provided in Table 7.

The MS results demonstrate that CA appears to form adducts with peptides containing positively charged amino acids, especially lysine and, to a lesser extent, arginine. This is observed, for example, in peptides No. 2, 3, 5, 6, 8, 10, 11, 12, 15, 16, and 17, which contain lysine residues, and in peptides No. 4 and 14, which contain arginine.

The ε-amino group of lysine (pKa approx. 10.5) and the guanidinium group of arginine (pKa approx. 12.5), remain protonated at pH 9 [49], suggesting that ionic interactions may initially mediate CA–peptide association. These interactions likely precede the oxidation of phenolate CA to CA–quinone, which then facilitates covalent bond formation. In fact, previous studies report that CA forms stable covalent adducts primarily with lysine, while no covalent adducts with arginine have been reported so far [9,48]. In agreement with this, the lysine-containing peptides identified in this study as adduct partners further support lysine’s role as a preferential nucleophilic binding site for CA under the applied conditions in the present study. Moreover, the MS data (Table 7) demonstrated that CA predominantly forms covalent adducts with β-Lg peptides in both its monomeric and dimeric forms. Notably, a higher number of putative CA dimer adducts (12 in total; Table 7, Figure 3, reaction products No. 5) was detected compared to putative CA monomer adducts (10 in total; Table 7), suggesting a possible preferential involvement of polymerized CA in peptide binding. This observation aligns with previous findings on the high polymerization tendency of *o*-dihydroxy PCs like CA via oxidative coupling [16]. Other studies further support this understanding, reporting that oxidized CA and related PCs, including CLA, form covalent adducts with β-Lg and other proteins, and that dimerization or oligomerization of the PC intermediates may increase their reactivity toward amino acid side chains [50]. Due to their larger and more complex structures, CA dimers exhibit increased reactivity than monomeric CA. Their enhanced resonance stabilization lowers the redox potential, thereby facilitating electron donation [51].

In contrast, the lower reactivity and structural flexibility of monomeric CA can be attributed to its higher redox potential and the reduced number of hydroxyl groups [48,51]. These characteristics may limit its ability to form multiple bonds or stable interactions, ultimately resulting in weaker binding affinity and a reduced tendency to form covalent adducts.

The finding that putative dimeric CA appears to bind covalently to peptides more frequently indicates that polymerization reactions of PCs can readily compete with the covalent binding of monomeric PCs. This insight contributes to the understanding of the dynamic reaction pathways of protein–phenol interactions, as outlined by Kieserling et al. (2024) [3].

Analogous to the incubation with CA, RA was also analyzed regarding its reactivity with β-Lg peptides. The DAD chromatograms revealed a decrease in peptide peak areas at retention times of 7, 8, and 11 min (Figure 5b, orange boxes) compared to the individual peptide mixture. Concurrently, new peaks appear at 5, 9, 10, 12, and 18 min (Figure 5b), green boxes), indicating the formation of reaction products.

The dominant peak at 16 min corresponds to monomeric RA, whose intensity notably decreases upon incubation with RA, suggesting its involvement in further reactions. This finding is supported by the appearance of additional incubation products at approx. 10 min in the RA blank. To further investigate these interactions, MS analysis was performed and the resulting adduct masses were summarized in Table 8. Shifts in the peptide retention times—attributed to adduct formation—are also included. The mass spectra confirm that RA forms covalent adducts with peptides as putative monomers, dimers, and trimers, which is in line with earlier findings on RA oligomerization by Ly et al. (2006) [42]. Specifically, 13 adducts were identified for putative monomeric RA based on exact masses (Figure 3, reaction products No. 6), eight for dimeric RA and seven for trimeric RA. Putative oligomeric RA preferentially binds to polar peptides (Figure 3, reaction product No. 7), as its structure contains an increased number of polar functional groups, such as carboxyl and hydroxyl groups, which enable hydrogen bonding and electrostatic interactions [48,49]. These interactions are assumed to be particularly favored with side chains of polar or charged amino acids like lysine, arginine, aspartic acid, or glutamic acid. This preference is reflected in the peptides involved in putative adduct formation with dimeric RA (peptides No. 6, 7, 11, 13, 16, and 19) and trimeric RA (peptides No. 5, 7, 9, and 13). These observations may appear to contrast with the findings of Bock et al. (2024), who reported a predominance of hydrophobic interactions between oligomeric RA and proteins [40]. However, based on the current data, it is hypothetically plausible that high-molecular-weight RA oligomers preferentially engage in hydrophobic interactions with nonpolar peptides, thereby reducing their accessibility for covalent bonding. In contrast, polar peptides could remain more sterically accessible in the absence of hydrophobically interacting RA, thereby enabling putative covalent adduct formation with monomeric RA.

Unlike its putative oligomeric forms, putative monomeric RA shows a broader binding profile, forming adducts with both polar peptides (No. 3, 5, 7, 11, 13, and 16) and nonpolar peptides (No. 1, 2, 4, 8, 10, and 17). This apparent difference to oligomeric RA may be related to its smaller molecular size and potentially greater structural flexibility, which facilitate steric accessibility of its hydrophobic regions. As a result, monomeric RA can also engage in hydrophobic interactions, allowing it to react with both polar and nonpolar peptides [48,49]. In its monomeric form, RA is less spatially constrained and can readily associate with the hydrophobic side chains of nonpolar amino acids such as valine or leucine.

Following the same approach as with CA and RA, CHL was also incubated with β-Lg peptides. The DAD chromatograms revealed a reduction in peptide peak areas upon incubation with CHL, particularly at retention times between 5 and 7, 11, and 13 min (Figure 5c, orange boxes), when compared to the peptide blank. Concurrently, new peaks appear at 6, 8, and 9 min (Figure 5c, green boxes), suggesting the formation of CHL–peptide interaction products. Notably, the chromatogram of incubated CHL in the absence of peptides (Figure 5c, CHL blank) displays a marked increase in signal intensity between 6 and 16 min, accompanied by the emergence of several new peaks (Figure 5c, C1–C6). This observation potentially indicates the formation of CHL-derived incubation products, likely originating from polymerization reactions. The longer retention times of these peaks are consistent with the formation of high-molecular-weight CHL oligomers or polymers.

To further investigate the formation of covalent CHL–peptide adducts, the incubated peptide samples were analyzed by MS. The results, summarized in Table 9, indicate that putative CHL monomeric, dimeric (Figure 3, reaction products No. 8), and cyclic dimeric forms may form covalent adducts with peptides. The formation of CHL dimers and cyclic dimers has previously been reported [27,48]. These studies describe peptide binding to cyclic CHL dimers, with cyclization proposed as a secondary reaction following initial conjugation, accompanied by elimination of water. In total, 13 adducts were detected for putative monomeric CHL, eight for putative dimeric CHL, and seven for putative cyclic dimeric CHL, all of which are listed individually in Table 9.

While monomeric CHL predominantly formed adducts with polar peptides, dimeric CHL also reacted with nonpolar peptides. This difference can be explained by their contrasting molecular properties. Monomeric CHL has a highly polar molecular character, with a Log P value of approx. −0.27 (calculated with ChemAxon), favoring hydrogen bonds and ionic interactions with polar peptides [52]. Under oxidative conditions, these interactions promote close spatial proximity, facilitating covalent adduct formation. In line with the findings of Tarahi et al. (2024), who identified preferential CHL binding at lysine−127 within a polar region of rice protein, our results further support the relevance of site-specific interactions in determining protein reactivity [53].

In contrast, CHL dimerization leads to increased hydrophobicity [27], which is reflected in an increase in the Log P value to up to 3.4 (calculated with ChemAxon). This increased hydrophobicity is likely due to intramolecular hydrogen bonding between the phenolic hydroxyl groups or carboxyl groups. Consequently, fewer free hydroxyl groups are available for interactions with water or polar peptides, thereby hypothetically increasing the relative contribution of hydrophobic interactions at the intermolecular level. The potential for hydrophobic interactions with non-polar peptides increases the likelihood of adduct formation under oxidative conditions due to spatial proximity.

Moreover, adduct formation of CHL is not limited to lysine and cysteine residues but also includes several other amino acids, such as tryptophan, histidine, and tyrosine [32,48,54]. However, the studies have shown that lysine tends to form adducts with CHL dimers. Furthermore, the observed cyclization of the dimer is primarily expected in the reaction with lysine but not with tryptophan, histidine, or tyrosine, as these amino acids lack the structural capability for cyclization [48].

Prigent et al. (2008) reported that tryptophan can form adducts with both monomeric and dimeric CHL, likely via a carbon atom in the pyrrole ring [48]. In the present study, peptides 4 and 6 contain tryptophan (Table 1). However, these peptides did not exhibit adduct formation with CHL, indicating that the reaction described by Prigent was not observed in the present work. Similarly, Prigent et al. (2008) described the formation of adducts with histidine, occurring via the imidazole ring [48]. In the present study, histidine is present in peptides No. 18 and 19. For peptide No. 18, adducts with monomeric and non-cyclized dimeric CHL were observed, supporting the involvement of the imidazole ring of histidine, particularly since no cyclization of CHL was found. Regarding tyrosine, Prigent et al. (2008) reported adduct formation primarily with CHL dimers but not with monomeric CHL [48]. In the present study, peptides No. 4, 5, 12, and 15 contain tyrosine. Adducts with monomeric CHL were not detected, whereas peptide No. 5 formed an adduct with a non-cyclized dimer, and peptides No. 12 and 15 formed adducts with cyclized dimers. As cyclization has not been described for tyrosine adducts, it seems to be probable that the binding site in peptides No. 12 and 15 is located at lysine.

The insights into the diverse reaction pathways of CHL closely reflect the challenges highlighted by Kieserling et al. (2024), who described that the interplay between covalent PC–peptide binding, PC polymerization reactions, and the influence of PC structure remains insufficiently understood [3].

## 3. Materials and Methods

### 3.1. Phenolic Compounds—Structural Characteristics

**Figure 9:**
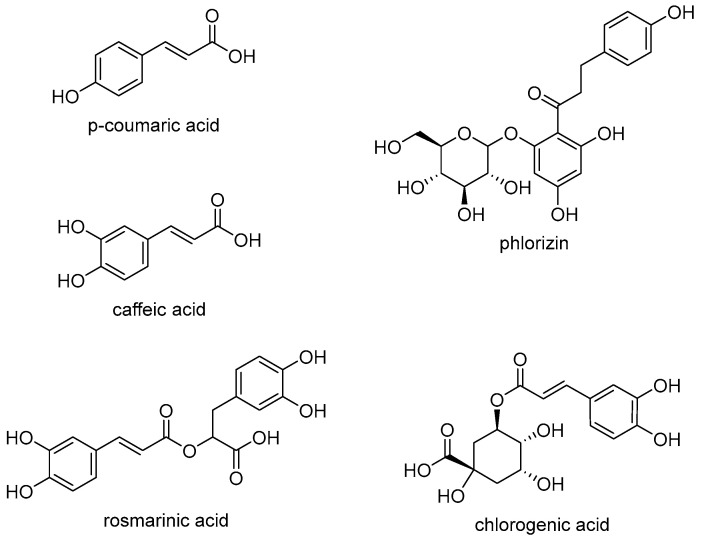
Chemical structures of the PCs used in the present study.

All selected PCs are based on either a hydroxycinnamic acid or a chalcone backbone, each exhibiting related but different substitution patterns (Figure 9). PCs used without glycosidic residues include *p*-coumaric acid (*p*-COU) and caffeic acid (CA). *p*-COU is a PC with a monohydroxy structure, bearing the hydroxyl group at the *p*-position relative to the side chain. CA is a PC with a dihydroxy structure, having hydroxyl groups in the *o*- and *p*- positions relative to each other (3,4-dihydroxy substitution). Another PC without glycosidic substituents is rosmarinic acid (RA), an ester of CA and 3,4-dihydroxyphenyllactic acid. Its structure contains two aromatic (benzene) rings, each substituted with two hydroxyl groups, connected via a propanoic acid-derived side chain and an ester linkage. Additionally, two PCs containing glycosidic or polar substituents were included: phlorizin (PHL) and chlorogenic acid (CHL). PHL is a natural glycoside composed of two phenyl rings linked by a reduced propenone-type side chain bearing a carbonyl group, with a glucose moiety attached to one ring. The A-ring (bearing the glucose unit) carries two hydroxyl substituents at the 2′- and 4′-positions (meta and para relative to the side chain), which are non-adjacent. The B ring (the second phenol ring) has a single hydroxyl group in the p-position. CHL, on the other hand, is the most prominent ester compound consisting of CA and quinic acid.

To prepare the PC solutions, they were weighed into a 20 mL-volumetric flask. 800 μL of ethanol were added for initial dissolving, followed by the addition of distilled water up to the mark. The final concentration of the PC solutions was 10.9 µmol/L. The PCs were purchased from Carl Roth GmbH & Co., KG (>98%; Karlsruhe, Germany).

### 3.2. β-Lactoglobulin Solutions and Tryptic Digestion

Native β-lactoglobulin (β-Lg) was isolated with a 99% purity from a commercial whey protein isolate (Davisco Foods International Inc., Minnesota, MN, USA), following the procedure described by Keppler et al. (2014) [55]. A mass of 200 mg β-Lg was diluted in phosphate buffer (pH 7.5; 0.1 M). The protein solution was stirred for 120 min at 20 °C for hydration.

For gaining peptides, a tryptic digestion was performed. A trypsin solution was prepared with 100 mg/100 mL in phosphate buffer (pH 7.5; 0.1 M). An aliquot of 4.9 mL of the β-Lg solution was transferred into a reaction vessel, to which 100 μL of the trypsin solution was added. The reaction vessel was then sealed and incubated under continuous shaking in a water bath at 37 °C for 16 h. To terminate the digestion, 1 mL of 1 M HCl (analytical grade, >99.9%, Carl Roth GmbH & Co., KG, Karlsruhe, Germany) was added.

### 3.3. Formation of Peptide–PC Adducts

The peptide mixture, resulting from the tryptic digestion of β-Lg, was mixed with the individual PC solutions (2.2) in a ratio of 1:1. The mixtures were then adjusted to pH 9.0 with 0.1 M NaOH (analytical grade, >99.9%, Carl Roth GmbH & Co., KG, Karlsruhe, Germany). The mixture was incubated under continuous shaking in a water bath at 20 °C for 16 h. For stopping the reaction (s), 1 mL of 1 M HCl (analytical grade, >99.9%, Carl Roth GmbH & Co., KG, Karlsruhe, Germany) was added. Three types of blank values were pure phosphate buffer, individual PC solutions (without peptides), and the peptide mixture with phosphate buffer (without PC). The blanks were also adjusted to pH 9 and incubated as described above.

### 3.4. Characterization of Covalent Adducts

The incubated peptide–PC mixtures and the blank values were analyzed for covalent adducts using HPLC–DAD and HPLC–MS in triplicate. These were carried out separately, but with the same chromatographic conditions; a gradient elution was utilized with eluent A consisting of 0.1% (*v*/*v*) formic acid (FA, ≥98%, Merck KGaA, Darmstadt, Germany) in water, and eluent B composed of 0.1% (*v*/*v*) FA in acetonitrile (≥99.0%, reagent grade, VWR International GmbH, Darmstadt, Germany). The gradient protocol was as follows for eluent B: 0–27 min: 6–53%, 27–29 min: 53–100%, 29–32 min: 100%, and 32–33 min: 100–6. Following the separation, a 4 min post-run phase was implemented to re-equilibrate the column to its initial conditions. Moreover, both systems applied the HPLC column PLRP-S (150 × 4.6 mm, 300 Å, 8 μm, Agilent Technologies Inc., Santa Clara, CA, USA).

For HPLC–DAD analysis, an Agilent 1220 Series HPLC system (Agilent Technologies Inc., Santa Clara, CA, USA) equipped with a diode array detector (DAD) a was used. Detection was carried out at 205 nm, specific for peptide detection, and 315 nm, specific for PC detection. Sample injection volume was 20 μL, with a flow rate of 1.0 mL/min and the column maintained at 30 °C.

For HPLC–MS analysis, an Agilent 1200 series HPLC system (Agilent Technologies Inc., Santa Clara, CA, USA) was used to determine the adduct masses. Following chromatographic separation, peptide characterization was performed using an LTQ Orbitrap XL™ mass spectrometer (Thermo Fisher Scientific Inc., Waltham, MA, USA). Each sample was analyzed in positive ionization mode within a mass range of 100–2000 *m*/*z* and 400–4000 *m*/*z*. Peptide identification was achieved by searching for specific amino acid sequences and the expected masses of their adducts. The analysis was conducted using FreeStyle™ 1.8 SP1 software (Thermo Fisher Scientific Inc., Waltham, MA, USA). The chemical properties of the resulting peptides were predicted using Expasy ProtParam 3.0 (SIB Swiss Institute of Bioinformatics, Lausanne, Switzerland, 2020).

## 4. Conclusions

This study contributes to the mechanistic understanding of the dynamic reaction pathways of PC oligomerization/polymerization, and peptide–PC adduct formation, building on well-established principles of PC oxidation and peptide reactivity and emphasizing how these reactions are determined by the chemical structure of the PC and the peptides. Oligomerization and polymerization of PCs appear to facilitate covalent peptide adduct formation, with reactivity patterns closely linked to hydroxylation and substituent pattern, thereby shedding light on the dynamic intermediate pathways that govern these reactions. Additional polar substituents promote interactions with polar amino acid side chains yet simultaneously impose steric hindrance that can limit polymerization and access to reactive peptide sites. The present findings demonstrate that the hydrophilic effect of polar substituents on PCs can be diminished by polymerization reactions, thereby altering potential binding sites. These observations underscore the intricate balance between molecular structure, reaction conditions such as pH, and the resulting reaction pathways determining peptide modification. By connecting molecular structure to reactivity patterns, this work provides mechanistic insights necessary to interpret and understand PC behavior in protein-rich food matrices. Given that extensive polymerization was observed, it remains unresolved whether this process competes with or supports covalent peptide binding. Future studies focusing on reaction kinetics will be essential to delineate the temporal relationships and relative contributions of oxidation, polymerization, and covalent modification, providing the foundation for understanding PC reactivity in food systems and guiding targeted applications in life sciences.

A detailed understanding of peptide binding sites represents a significant advance, as it enables precise control of covalent protein–polyphenol interactions, including epitope masking and the modulation of functional properties. These insights open avenues for the targeted design of protein-rich food systems with improved texture, stability, and potentially reduced allergenicity.

## Figures and Tables

**Table 1:** Characteristics of the tryptic peptides derived from β-lactoglobulin. Predicted chemical properties for each peptide were calculated using the ExPASy ProtParam 3.0 tool, including the Grand Average of Hydropathicity (GRAVY) value, the theoretical isoelectric points (IEPs), the molecular weight (MW) and the retention time (RT).

Peptide No.	Amino Acid Sequence	GRAVY	IEP	MW [*m*/*z*]	RT_DAD_ [min]	RT_MS_ [min]
1	MKCLLLALALTCGAQA	1.538	7.82	1620.06	13.6	14.4
2	LIVTQTMK	0.700	8.75	933.17	8.6	9.6
3	GLDIQK	−0.500	5.84	672.78	n.a.	7.1
4	VAGTWYSLAMAASDISLLDAQSAPLR	0.519	4.21	2708.08	n.a.	25.6
5	VYVEELKPTPEGDLEILLQK	−0.315	4.25	2313.67	n.a.	26.0
6	WENGECAQK	−1.656	4.53	1064.14	17.3	18.5
7	K	n.a.	9.74	146.19	n.a.	n.a.
8	IIAEK	0.680	6.00	572.70	3.3	4.3
9	TK	n.a.	n.a.	247.29	n.a.	n.a.
10	IPAVFK	1.300	8.75	673.85	10.4	11.6
11	IDALNENK	−0.975	4.37	916.00	5.6, 5.9	6.6
12	VLVLDTDYK	0.344	4.21	1065.23	11.8	12.5
13	K	n.a.	9.74	146.19	n.a.	n.a.
14	YLLFCMENSAEPEQSLACQCLVR	0.226	4.25	2648.08	16.8	17.2
15	TPEVDDEALEK	−1.264	3.83	1245.31	8.2	8.9
16	FDK	n.a.	n.a.	408.44	2.6	3.5
17	ALK	n.a.	n.a.	330.41	n.a.	n.a.
18	ALPMHIR	0.386	9.80	837.05	n.a.	20.7
19	LSFNPTQLEEQCHI	−0.457	4.51	1658.85	n.a.	25.9

n.a. = not applicable.

**Table 2:** Peptide–PC adduct masses of *p*-COU and β-Lg peptides and their retention times (RTs, detected as proton adducts).

	Adduct Mass [*m/z*]^+^ (=M_peptide_ + M_p-COU_ − 2H)		Original RT [min]	Potentially Shifted RT [min]
	Initial Peptide Characteristics			
Peptide No.	Nonpolar	Polar		
1	n.a.	n.a.	n.a.	n.a.
2	1095.5924	n.a.	9.6	15.5
3	n.a.	835.4348	7.1	14.9
4	2869.4326	n.a.	25.6	22.6
5	n.a.	n.a.	n.a.	n.a.
6	n.a.	n.a.	n.a.	n.a.
7	n.a.	n.a.	n.a.	n.a.
8	735.4102	n.a.	4.3	19.1
9	n.a.	n.a.	n.a.	n.a.
10	836.4687	n.a.	11.6	17.5
11	n.a.	1078.5166	6.6	7.1
12	1227.6237	n.a.	12.5	11.5
13	n.a.	n.a.	n.a.	n.a.
14	2809.2527	n.a.	17.2	18.5–34.2
15	n.a.	n.a.	n.a.	n.a.
16	n.a.	571.3024	3.5	12.9
17	n.a.	n.a.	n.a.	n.a.
18	n.a.	n.a.	n.a.	n.a.
19	n.a.	n.a.	n.a.	n.a.
M_p-COU_	164.04373		n.a.	13.4

n.a. = not applicable.

**Table 3:** Peptide–PC adduct masses of PHL and β-Lg peptides and their retention times (RTs, detected as proton adducts).

	Adduct Mass [*m/z*]^+^ (=M_peptide_ + M_PHL_ − 2H)		Original RT [min]	Potentially Shifted RT [min]
	Peptide Characteristics			
Peptide No.	Nonpolar	Polar		
1	2054.2239	n.a.	14.4	13.2
2	n.a.	n.a.	n.a.	n.a.
3	n.a.	1107.7913	7.1	24.8
4	n.a.	n.a.	n.a.	n.a.
5	n.a.	2747.6589	26.0	21.9
6	n.a.	1498.8547	18.5	16.4
7	n.a.	n.a.	n.a.	n.a.
8	n.a.	n.a.	n.a.	n.a.
9	n.a.	682.5577	n.a.	9.1–26.8
10	n.a.	n.a.	n.a.	n.a.
11	n.a.	n.a.	n.a.	n.a.
12	1499.9841	n.a.	12.5	16.5–21.5
13	n.a.	n.a.	n.a.	n.a.
14	3081.6091	n.a.	17.2	16.7
15	n.a.	n.a.	n.a.	n.a.
16	n.a.	843.6095	3.5	6.4
17	n.a.	n.a.	n.a.	n.a.
18	n.a.	n.a.	n.a.	n.a.
19	n.a.	n.a.	n.a.	n.a.
M_PHL_	437.1439			14.7

n.a. = not applicable.

**Table 4:** Masses corresponding to peaks in the chromatograms after incubation of CA with retention times (RTs), expected and theoretical values, and deviations in ppm (*m*/*z* as proton adducts).

Peak	RT_MS_ [min]	M_exp._ [*m/z*]^+^	M_theor._ [*m/z*]^+^	Delta [ppm]	Putative Compound
A1	10.20	547.0840	n.a.	n.a.	CA trimer with modifications
		1088.1510	n.a.	n.a.	CA hexamer with modification
A2	12.38–16.09	177.0199	177.0194	2.8245	CA monomer (as quinone)
A3	14.51	359.0758	359.0761	0.8354	CA dimer
		479.0757	n.a.	n.a.	CA dimer or trimer with modifications
A4	15.23	359.0759	359.0761	0.5570	CA dimer
		479.0757	n.a.	n.a.	CA dimer or trimer with modifications
A5	15.46	359.0757	359.0761	0.2785	CA dimer
A6	19.33	539.1184	539.1211	5.0081	CA trimer

n.a. = not applicable.

**Table 5:** Masses corresponding to peaks in the chromatograms after incubation of RA with retention times (RTs), expected and theoretical values, and deviations in ppm (*m*/*z* as proton adducts).

Peak	RT_MS_ [min]	M_exp._ [*m*/*z*]^+^	M_theor._ [*m*/*z*]^+^	Delta [ppm]	Putative Compound
B1	6.38	717.1815	717.1814	−0.1394	RA dimer
B2	13.96–23.64	539.1180	539.1184	0.7420	RA–CA adduct
B3	15.97	317.1011	317.1020	2.8382	Teucrol
B4	17.18	361.0916	361.0918	0.5539	RA monomer
	17.16	721.1757	721.1752	−0.6933	RA dimer
	17.16–17.30	1079.2460	1079.2441	−1.7605	RA trimer
	17.13–17.22	1441.3451	1441.3421	−2.0814	RA tetramer
	17.16	1800.4113	1800.4182	3.8324	RA pentamer

**Table 6:** Masses corresponding to peaks in the chromatograms after incubation of CHL with retention times (RTs), expected and theoretical values, and deviations in ppm (*m*/*z* as proton adducts).

Peak	RT_MS_ [min]	M_exp._ [*m*/*z*]^+^	M_theor._ [*m*/*z*]^+^	Delta [ppm]	Putative Compound
C1	6.44	355.1021	355.1024	0.8448	CHL monomer
C2	8.11–8.42	355.1021	355.1024	0.8448	CHL monomer
C3	10.40–10.69	707.1806	707.1818	1.6969	CHL dimer
	10.46	705.1649	705.1661	1.7017	CHL dimer, cyclic
C4	11.63	707.1807	707.1818	1.5555	CHL dimer
C5	13.04	533.1285	n.a.	n.a.	CHL dimer with modifications
C6	14.59	456.1168	n.a.	n.a.	CHL dimer with modifications

n.a. = not applicable.

**Table 7:** Peptide–PC adduct masses of CA and β-Lg peptides and their retention times (RTs, detected as proton adducts).

	Adduct Mass [*m/z*]^+^ (=M_peptide_ + M_CA−2H_)		Original RT_MS_ [min]	Potentially Shifted RT_MS_ [min]	Adduct Mass [*m/z*]^+^ (=M_peptide_ + M_2×CA−4H_)		Original RT_MS_ [min]	Potentially Shifted RT_MS_ [min]
Peptide	Nonpolar	Polar			Nonpolar	Polar		
1	1798.2048	n.a.	14.4	8.59	n.a.	n.a.	n.a.	n.a.
2	1111.6990	n.a.	9.6	10.67	1289.6125	n.a.	9.6	16.73
3	n.a.	851.5459	7.1	11.44	n.a.	1029.4604	7.1	7.53
4	n.a.	n.a.	n.a.	n.a.	3036.4360	n.a.	25.6	12.07
5	n.a.	n.a.	n.a.	n.a.	n.a.	n.a.	n.a.	n.a.
6	n.a.	1042.5319	18.5	12.15	n.a.	1420.4955	18.5	9.42
7	n.a.	n.a.	n.a.	n.a.	n.a.	n.a.	n.a.	n.a.
8	n.a.	n.a.	n.a.	n.a.	929.4304	n.a.	4.3	10.24–19.19
9	n.a.	n.a.	n.a.	n.a.	n.a.	n.a.	n.a.	n.a.
10	852.6366	n.a.	11.6	16.67	1030.4845	n.a.	11.6	13.74
11	n.a.	1094.6332	6.6	7.02	n.a.	1272.5353	6.6	7.50–21.34
12	1243.7330	n.a.	12.5	7.02	n.a.	n.a.	n.a.	n.a.
13	n.a.	n.a.	n.a.	n.a.	n.a.	n.a.	n.a.	n.a.
14	2825.3457	n.a.	17.2	26.01	3003.2693	n.a.	17.2	14.34
15	n.a.	n.a.	n.a.	n.a.	n.a.	1601.6439	8.9	11.96
16	n.a.	n.a.	n.a.	n.a.	n.a.	765.2756	3.5	5.65–17.45
17	509.3890	n.a.	n.a	7.11	687.2969	n.a.	n.a	12.07–18.77
18	n.a.	n.a.	n.a.	n.a.	n.a.	n.a.	n.a.	n.a.
19	n.a.	1836.9390	25.9	5.47	n.a.	2014.8451	25.9	15.90
M_CA-quinone_	177.0197							6.64–8.79

n.a. = not applicable.

**Table 8:** Peptide–PC adduct masses of RA and β-Lg peptides and their retention times (RTs, detected as proton adducts).

	Adduct Mass [*m/z*]^+^ (=M_peptide_ + M_RA−2H_)		Original RT_MS_ [min]	Pot. Shifted RT [min]	Adduct Mass [*m/z*]^+^ (=M_peptide_ + M _2×RA−2H_)		Original RT_MS_ [min]	Pot. Shifted RT [min]	Adduct Mass [*m/z*]^+^ (=M_peptide_ + M_3×RA−2H_)		Original RT_MS_ [min]	Pot. Shifted RT [min]
Peptide No.	Nonpolar	Polar			Nonpolar	Polar			**nonpolar**	**polar**		
1	1979.1447	n.a.	14.4	21.3	n.a.	n.a.	n.a.	n.a.	n.a.	n.a.	n.a.	n.a.
2	1292.6310	n.a.	9.6	9.2	n.a.	n.a.	n.a.	n.a.	n.a.	n.a.	9.6	8.3
3	n.a.	1032.4800	7.1	20.9	n.a.	n.a.	n.a.	n.a.	n.a.	n.a.	n.a.	n.a.
4	3066.4714		n.a.	n.a.	n.a.	n.a.	n.a.	n.a.	n.a.	n.a.	n.a.	n.a.
5	n.a.	22.26	n.a.	n.a.	n.a.	n.a.	n.a.	n.a.	n.a.	3390.5076	n.a.	12.2
6	n.a.	n.a.	n.a.	n.a.	n.a.	1779.6382	18.5	12.29–12.84	n.a.	n.a.	n.a.	n.a.
7	n.a.	506.2024	n.a.	17.4	n.a.	862.2908	n.a	16.76	n.a.	1224.3535	18.5	17.1
8	932.4530	n.a.	4.3	12.4–22.5	1288.5459		4.3	20.14	n.a.	n.a.	n.a.	n.a.
9	n.a.	n.a.	n.a.	n.a.	n.a.	n.a.	n.a.	n.a.	n.a.	1325.4041	4.3	20.7 −20.9
10	1033.5137	n.a.	11.6	24.9	n.a.	n.a.	n.a.	n.a.	1751.6683	n.a.	n.a.	16.2 −16.3
11	n.a.	1275.5621	6.6	4.1	n.a.	1631.6575	6.6	22.32	n.a.	n.a.	n.a.	n.a.
12	n.a.	n.a.	n.a.	n.a.	1780.7589	n.a.	12.5	11.83	n.a.	n.a.	n.a.	n.a.
13	n.a.	506.2024	n.a	17.4	n.a.	862.2908	n.a	16.76	n.a.	1224.3535	12.5	17.1
14	n.a.	n.a.	n.a.	n.a.	n.a.	n.a.	n.a.	n.a.	n.a.	n.a.	n.a	n.a.
15	n.a.	n.a.	n.a.	n.a.	n.a.	n.a.	n.a.	n.a.	n.a.	n.a.	n.a.	n.a.
16	n.a.	768.3024	3.5	7.6	n.a.	1124.3964	3.5	11.32	n.a.	n.a.	n.a.	n.a.
17	690.3228	n.a.	n.a	18.7–20.6	n.a.	n.a.	n.a.	n.a.	1408.4753	n.a.	3.5	21.7
18	n.a.	n.a.	n.a.	n.a.	n.a.	n.a.	n.a.	n.a.	n.a.	n.a.	n.a.	n.a.
19	n.a.	n.a.	n.a.	n.a.	n.a.	2373.9618	25.9	9.40	n.a.	n.a.	n.a.	n.a.
M_RA_												17.2

n.a. = not applicable.

**Table 9:** Peptide–PC adduct masses of CHL and β-Lg peptides and their retention times (RT, detected as proton adducts).

	Adduct Mass [*m/z*]^+^ (=M_peptide_ + M_CHL−2H_)		Original RT_MS_ [min]	Pot. Shifted RT [min]	Adduct Mass [*m/z*]^+^ (=M_peptide_ + M_2×CHL−2H_)		Original RT_MS_ [min]	Pot. Shifted RT [min]	Adduct Mass [*m/z*]^+^ (=M_peptide_ + M_2×CHL, cycl.−2H_)		Original RT_MS_ [min]	Pot. Shifted RT [min]
Peptide No.	Nonpolar	Polar			Nonpolar	Polar			Nonpolar	Polar		
1	n.a	n.a	n.a	n.a	n.a	n.a	n.a	n.a	2323.2161	n.a	14.4	16.3
2	n.a	n.a	n.a	n.a	1638.7141	n.a	9.6	17.3	1636.7076	n.a	9.6	10.1–17.9
3	n.a	1026.4930	7.1	9.6; 16.2	n.a	13.5424	7.1	4.8	n.a	1376.5588	7.1	19.1
4	n.a	n.a	n.a	n.a	n.a	n.a	n.a	n.a	n.a	n.a	n.a	n.a
5	n.a	n.a	n.a	n.a	n.a	3018.4565	26.0	22.6	n.a	n.a	n.a	n.a
6	n.a	n.a	n.a	n.a	n.a	n.a	n.a	n.a	n.a	n.a	n.a	n.a
7	n.a	500.2122	n.a	9.1–12.8	n.a	852.2927	n.a	15.9–17.6	n.a	850.2761	n.a	6.3
8	926.4523	n.a	4.3	20.3	1278.5418	n.a	4.3	18.6	1276.5227	n.a	4.3	14.6
9	n.a	601.2634	n.a.	5.2; 11.8	n.a	953.3428	n.a.	18.5		951.3271	n.a.	19.5
10	1027.5257	n.a	11.6	16.9–17.9	1379.5995	n.a	11.6	12.4; 17.2	1377.5861	n.a	11.6	17.4–21.5
11	n.a	1269.5715	6.6	8.5–8.6	n.a	n.a	n.a	n.a	n.a	1619.6305	6.6	23.9
12	n.a	n.a	n.a	n.a	n.a	n.a	n.a	n.a	1768.7405		12.5	25.9
13	n.a	500.2122	n.a	9.1–12.8	n.a	852.2927	n.a	15.9–17.6		850.2761	n.a	6.3
14	n.a	n.a	n.a	n.a	n.a	n.a	n.a	n.a	3350.3818	n.a	17.2	23.3
15	n.a	n.a	n.a	n.a	n.a	1950.7694	8.9	14.8–16.2	n.a	n.a	n.a	n.a
16	n.a	726.3128	3.5	11.4	n.a	1114.3912	3.5	9.5–9.9	n.a	1112.3787	3.5	11.0
17	684.3307	n.a	n.a	6.1	1036.4121	n.a	n.a	17.6–18.9	1034.3961	n.a	n.a	16.9
18	1190.5847	n.a	20.7	19.7	1542.6652	n.a	20.7	9.9	n.a	n.a	n.a	n.a
19	n.a	n.a	n.a	n.a	n.a	n.a	n.a	n.a	n.a	n.a	25.9	n.a
M_CHL_			355.1017									6.4

n.a. = not applicable.

## Data Availability

Dataset available on request from the authors.
